# Self-Healing Efficiency of Cementitious Materials Containing Microcapsules Filled with Healing Adhesive: Mechanical Restoration and Healing Process Monitored by Water Absorption

**DOI:** 10.1371/journal.pone.0081616

**Published:** 2013-11-28

**Authors:** Wenting Li, Zhengwu Jiang, Zhenghong Yang, Nan Zhao, Weizhong Yuan

**Affiliations:** 1 Key Laboratory of Advanced Civil Engineering Materials of Ministry of Education, Department of Materials Science and Engineering, Tongji University, Shanghai, People’s Republic of China; 2 Department of Materials Science and Engineering, Tongji University, Shanghai, People’s Republic of China; Faculdade de Medicina Dentária, Universidade do Porto, Portugal

## Abstract

Autonomous crack healing of cementitious composite, a construction material that is susceptible to cracking, is of great significance to improve the serviceability and to prolong the longevity of concrete structures. In this study, the St-DVB microcapsules enclosing epoxy resins as the adhesive agent were embedded in cement paste to achieve self-healing capability. The self-healing efficiency was firstly assessed by mechanical restoration of the damaging specimens after being matured. The flexural and compressive configurations were both used to stimulate the localized and distributed cracks respectively. The effects of some factors, including the content of microcapsules, the curing conditions and the degree of damage on the healing efficiency were investigated. Water absorption was innovatively proposed to monitor and characterize the evolution of crack networks during the healing process. The healing cracks were observed by SEM-EDS following. The results demonstrated that the capsule-containing cement paste can achieve the various mechanical restorations depending on the curing condition and the degree of damage. But the voids generated by the surfactants compromised the strength. Though no noticeable improved stiffness obtained, the increasing fracture energy was seen particularly for the specimen acquiring 60% pre-damage. The sorptivity and amount of water decreased with cracks healing by the adhesive, which contributed to cut off and block ingress of water. The micrographs by SEM-EDS also validated that the cracks were bridged by the hardened epoxy as the dominated elements of C and O accounted for 95% by mass in the nearby cracks.

## Introduction

Cracks, due to various reasons as legacy of poor construction methods, quality controls, can have a great influence on safety and durability of concrete structures, in terms of load resistivity and transport properties mostly [1-7]. Repair and maintenance costs have risen rapidly within these decades, particularly in the developed countries to ensure reliability and to prolong longevity of concrete structures. 

Cracks can be passively repaired with a deliberate external intervention once cracking is monitored. But there are still many conditions where repair may be difficult or impossible to be executed, for instance, too fine or too deep-embedded damage, and infrastructures in continuous service. In these cases, the bio-inspired materials, like natural healing of wounds or cuts in living species, are of great interests since they can be autonomic-sensing, autonomic-repairing, or rather self-healing to restore their structural integrity[8]. Inspired by this, many scientists have tried to creatively incorporate this idea into engineering artificial materials like cementitious composite to make it intelligent [9-20] since it was originally proposed for cement matrix by Dry [21]. This is highly beneficial for improving the serviceability of civil constructions even though there are some challenges, whereby the healing efficiency and underlying mechanisms are the keys to achieve this.

By now the healing efficiency has been highlighted mostly by liquid tightness or transfer properties of concrete, initially deteriorated by the presence of cracks which becomes finer after healing [22-30]. Nearly all the automatic healing, like crystallization of mineral additives, polymers, or additional hydration of unhydrated cementitious components etc., in cementitious composites have demonstrated this effect remarkably since the cracks can be easily filled to prevent the open paths for liquids. Besides, an improvement of the mechanical performance should be another major consequence of concern. However, only polymers as a healing agent stored in capsules or tubes have shown a relatively rapid recovery of the mechanical properties of host materials. 

Encapsulation techniques have been demonstrated to be a promising solution for cracking as highlighted by an improvement of mechanical performance and/or transport properties of material. Though the improvement of mechanical behavior has been more reported on the other host matrix as polymers [31-40], woven composites [41], or fiber reinforced composites [42], the mechanical recovery of self-healing cementitious composites containing microcapsules has become an increasing attention [43-48]. Pelletier et al. [43] reported 26% of the original load resistance of a self-healing concrete system involving internal encapsulation of a sodium silicate solution in polyurethane microcapsules present in the matrix, compared to a recovery of 10% of the references without capsules. Besides the strength recovery, the improved toughness and the attenuation of corrosion have also been obtained. The work of Nishiwaki [44-46] demonstrated that the insufficient mixing of the two part-released resins from the capsules, i.e. the healing agent and the catalyst, can result in a poor polymerization degree and therefore poor mechanical performance of the adhesive in terms of compressive strength and splitting strength. Yang et al. [47] obtained 45.8% and 30.4% increase in compressive strength of the mortar at 1d and 28d age respectively, incorporating the microcapsules with methylmethacrylate monomer and triethylborane as the healing agent and the catalyst for use in the cementitious system. Hu et al. [48] developed a cement paste involving urea formaldehyde (UF) microcapsules filled with epoxy resins to exhibit self-healing properties. They evaluated the healing efficiency by means of the compressive strength recovery of the healing specimen to that of the intact one. They found that the healing efficiency can reach to 111% for the paste with 1% of microcapsules and 60% pre-damage, defined as the ratio of the load applied to the resistance of the intact specimen, loaded in the same fashion. The role of self healing in mechanical properties of cementitious composites is thus a major concern of the present study.

Furthermore, crack healing by the adhesive in capsules is such a process, including release of the adhesive upon breakage, its polymerization and a gain of the strength. The properties of cementitious composite, other than the mechanical property, can be affected by this process as the cracks being filled in, and then narrowed until closed gradually. However, no work has been reported on this healing process but instead, only the changes, like mechanical performance and/or permeability, in state have been tracked by now. To do this, water absorption is proposed to monitor and characterize the evolution of crack networks and voids in the healing process based on that the capillary effect, as the prime driving force for the sucktion of water of partially wetted porous materials like cementitious composite [49,50], is closely related to the characteristics of microcrack system, such as crack geometry and connectivity.

## Experimental Program

### Materials

Diglycidylether of bisphenol A epoxy resin (E) and poly styrene-divinylbenzene (St-DVB) were chosen as the core material and the wall respectively for use. E was commercially available from Ltd. of Shanghai Chenyi Chemicals, China. Benzylalcohol (BA) was used as the reactive diluents of St-DVB. Monomers styrene (St), divinylbenzene (BPO) and benzoylperoxide (BP) as the trigger of polymerization were commercially available as well. Additional surfactants as sodium dodecylbenzene sulfonate (SDBS), octylphenol ethoxylate (OP-10) and potassium peroxydisulfate (PP) were also obtained for encapsulation. All the solvents were of analytical grade and supplied by Sinopharm Chemical Reagent Co., Ltd, China. The amine hardener of epoxy from Shanghai Hanzhong Chemicals Co., Ltd, China was used in the matrix.

All the paste specimens were made with ordinary Portland cement (Type I) and tap water from the lab.

### Preparation of microcapsules

**Figure 1:**
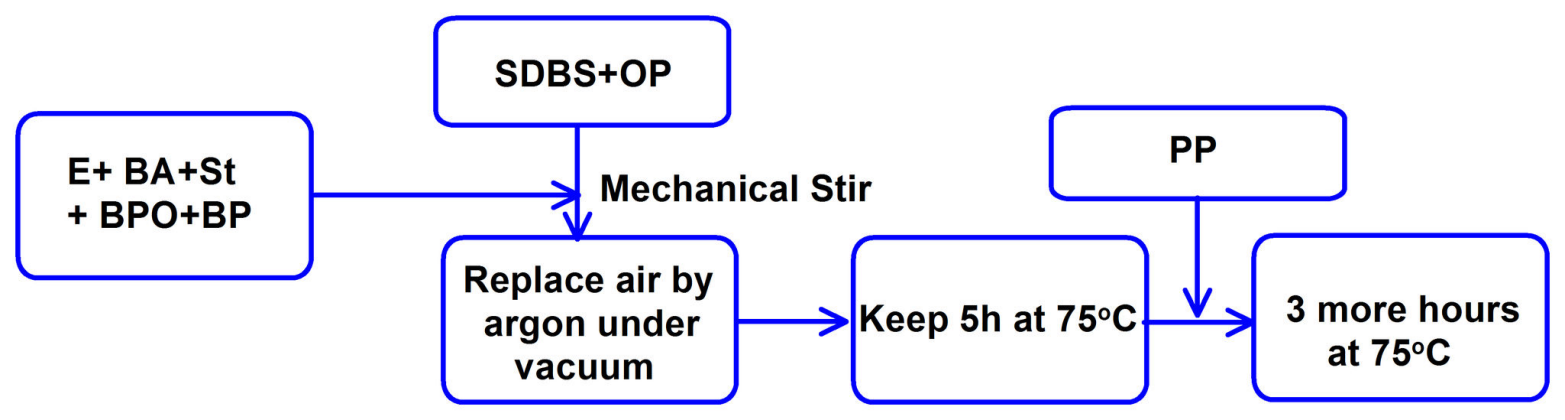
Preparation procedure of microcapsules.

The epoxy was firstly mixed with BA (14 wt%) in a 500-ml three-neck round-bottomed flask for dilution. The surfactant water solution of SDBS (4 wt%) and OP (4 wt%) with an amount of 2.8 times of the epoxy was added into the flask and mechanically stirred to form a well-dispersed solution. The solution was gradually heated up to 75°C and maintained at this temperature for 1h. While still under this temperature, the mixture of St, BPO and BP was then added into this solution. After that, air in the container was replaced by argon through an opening on the flask with the help of a vacuum pump. The temperature was maintained for 5h for synthesis. PP (1 wt%) was then added and allowed for 3 more hours for continuous reaction. An emulsion of microcapsules with 30% concentration by mass was obtained finally. The preparation procedure is shown in Figure 1.

### Preparation of self-healing cement paste containing microcapsules

Cement paste with 0.3 w/c was used as the host matrix throughout. The hardener was added when mixing by the ratio of 1.3 times of the epoxy. The healing efficiency was considered, while varying the content of microcapsules from 0% till 2% of cement by mass. For each content, the specimens were prepared into two batches for standard curing (20°C±2°C, ≥95%RH) and water curing respectively. For each condition, 3 groups were needed for the various levels of pre-damage, referring to 30%, 60% and 100% of the maximum resistance *f*. Three specimens for each group were used to get the results in average.

### Testing procedures

The morphology of microcapsules was observed by OM.

**Figure 2:**
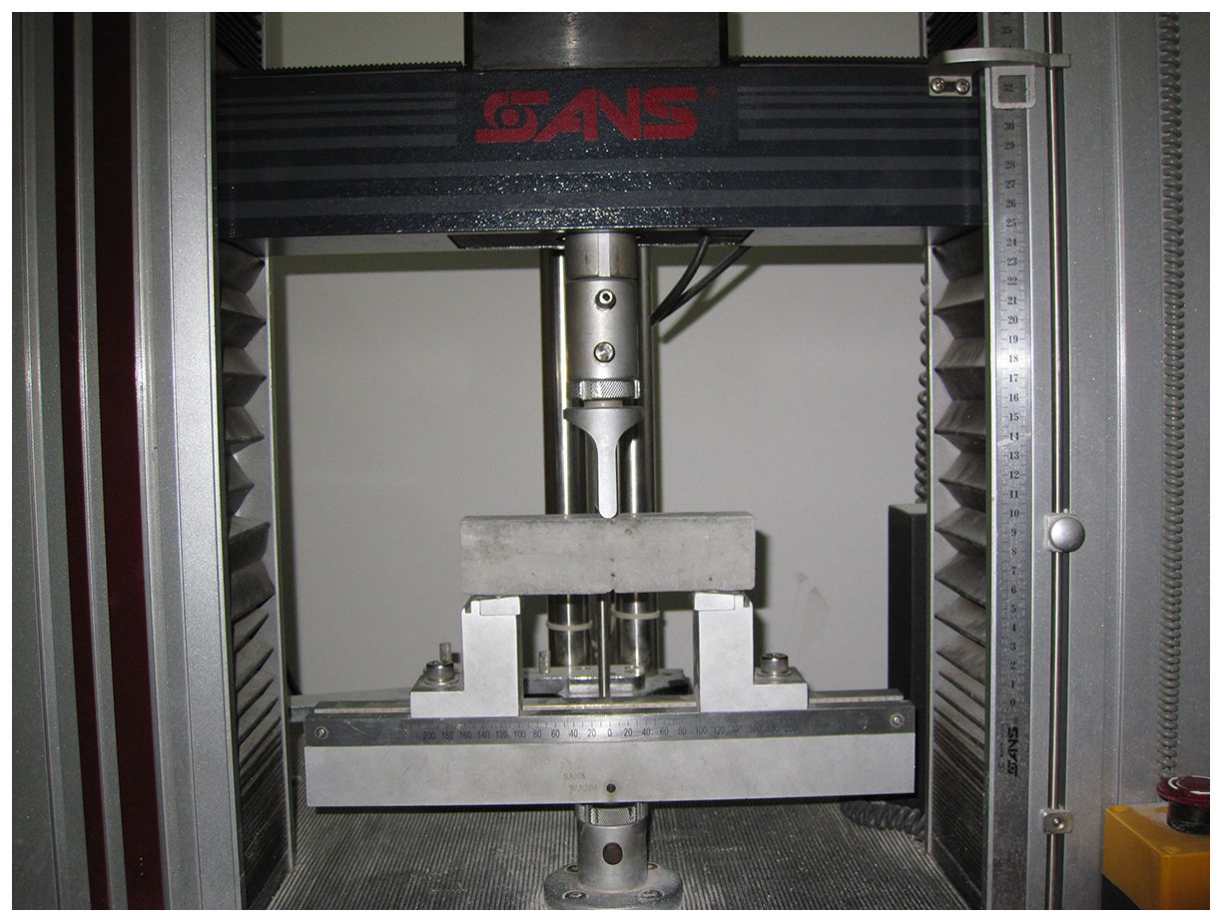
Three-point flexural loading setup.

Two specimen geometries were used in this study: cylinders (25mm height × 100mm diameter) and prisms (40mm × 40mm × 160mm). Cylindrical specimens were used for the water absorption test according to ASTM C1585-04 [51]. The prism specimens were used for flexural strength following ASTM C348 [52]; besides, a notch was fabricated to make stress-intensive effect referring to RILEMTC50-FMC [53] about using three-point bend tests of notched beams to determine the fracture energy of mortar and concrete. But the size of notch was proportionally decreased to 10mm length by 0.5mm thick for the nonstandard prisms. One group of the prisms was firstly loaded under 3-point flexural configuration until failure during which the complete load-displacement curve was recorded. The maximum load bearing *f* was determined accordingly. The second group was loaded until 30%*f* and then unloaded to generate cracks. Following that, one day was allowed for the damaging specimen to heal autonomously. The healing specimen was reloaded again until its failure under exactly the same conditions and the load-displacement curve was measured. The same experimental procedures were executed on the specimens by 60%*f*. The 3-point flexural loading setup was from Shanghai Songdun Mechanical Equipment Co. Ltd. shown in Figure 2.

The two parts from the failed specimens were cut into cubic (40mm length) to conduct the compression tests, by which the distributed cracks can be induced. The strength recovery was measured, following the procedure of that by flexural configuration. 

**Figure 3:**
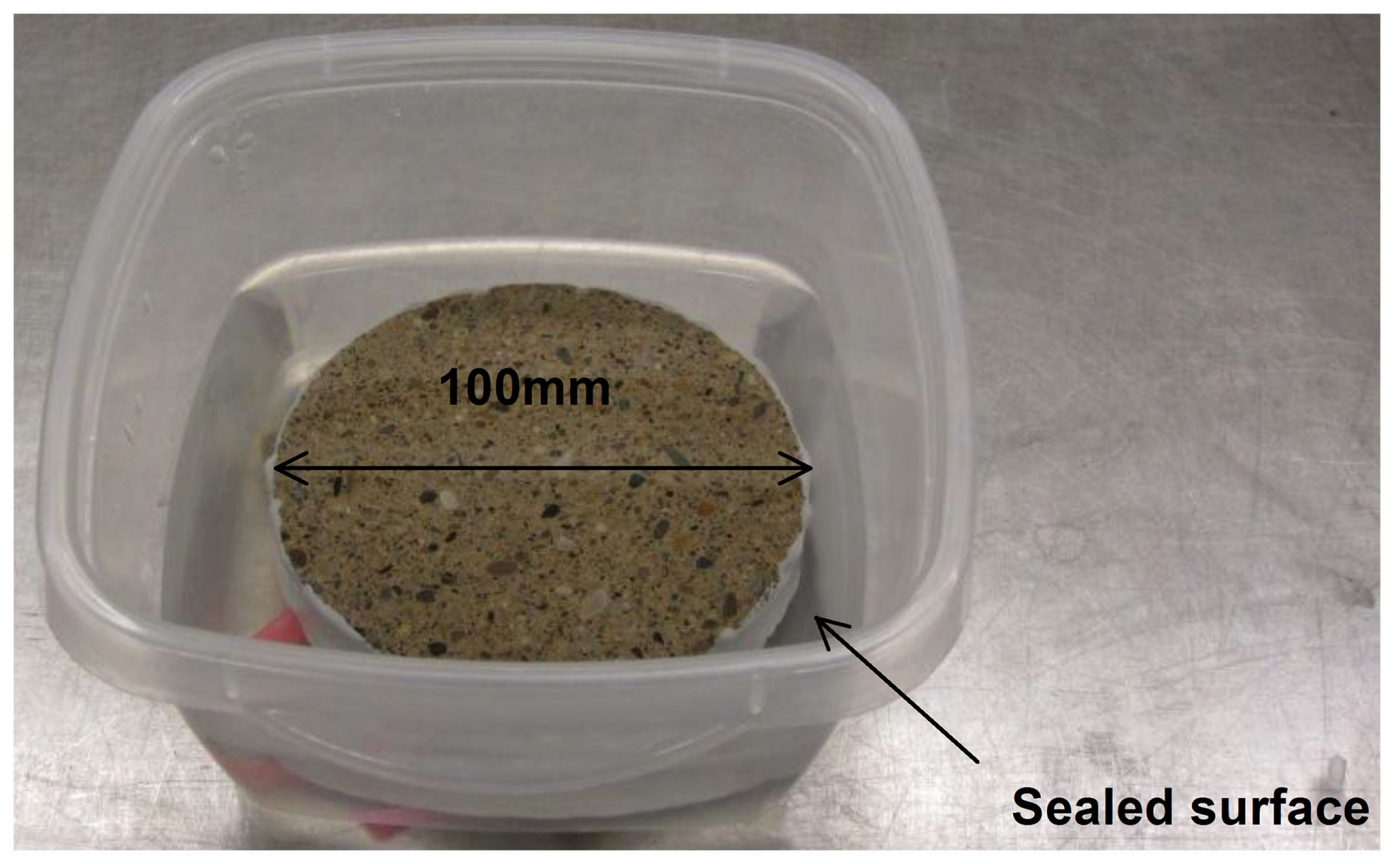
Specimen preparations for water absorption.

A procedure similar to ASTM C1585-04 [51] was used for water absorption, as shown in Figure 3.

Pieces of the damaged specimens were taken out and observed using SEM-EDS at the different stages of healing, referring to 30%*f*, 60%*f* and 100%*f*. 

## Results and Discussion

**Figure 4:**
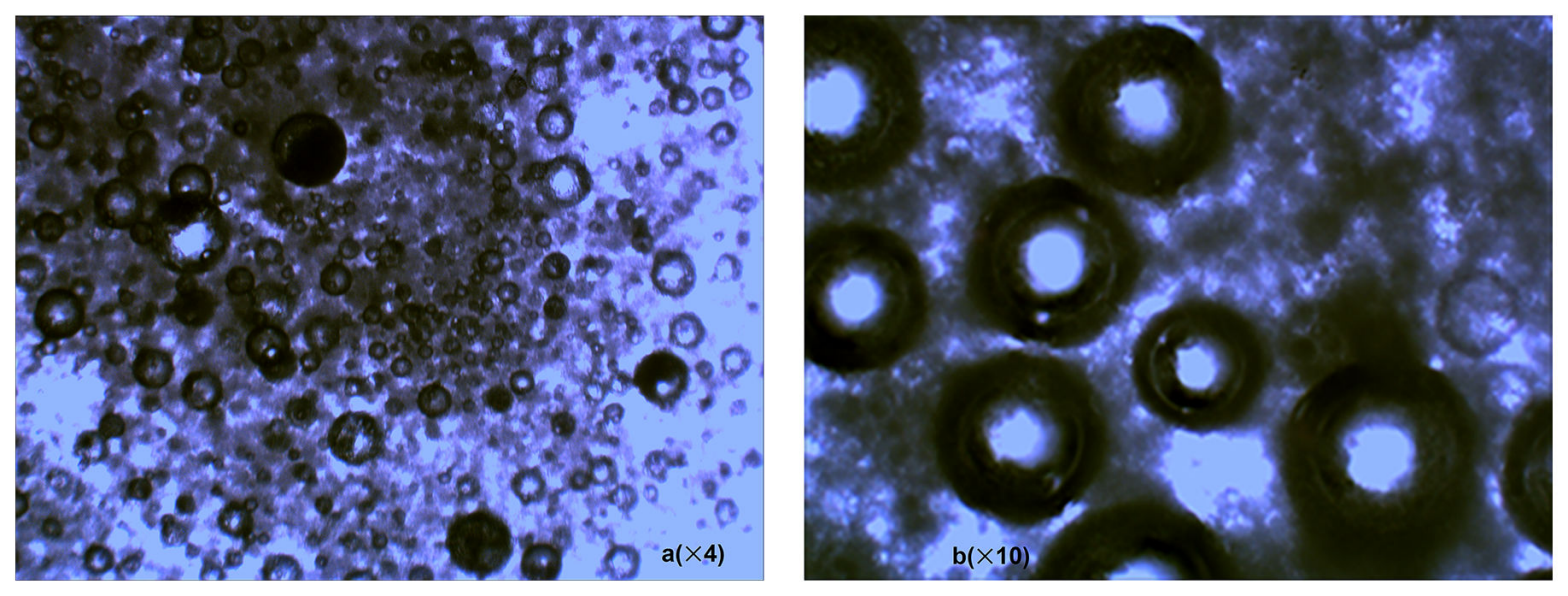
OM micrographs of microcapsules prepared: (a) ×4 (b) ×10.

Figure 4 shows the OM micrographs of the microcapsules prepared. A great many spherical capsules were evenly distributed with a regular shape as shown in Figure 4a. The capsules were compact and with the size mostly in between 100-150µm. It displayed two layers clearly under a higher magnification as shown in Figure 4b, including a core droplet (light area) and a wall shell (dark area) that account for 1/3 and 2/3 of the capsule in diameter respectively. The floccus filled in between the capsules were residual surfactants. 

**Figure 5:**
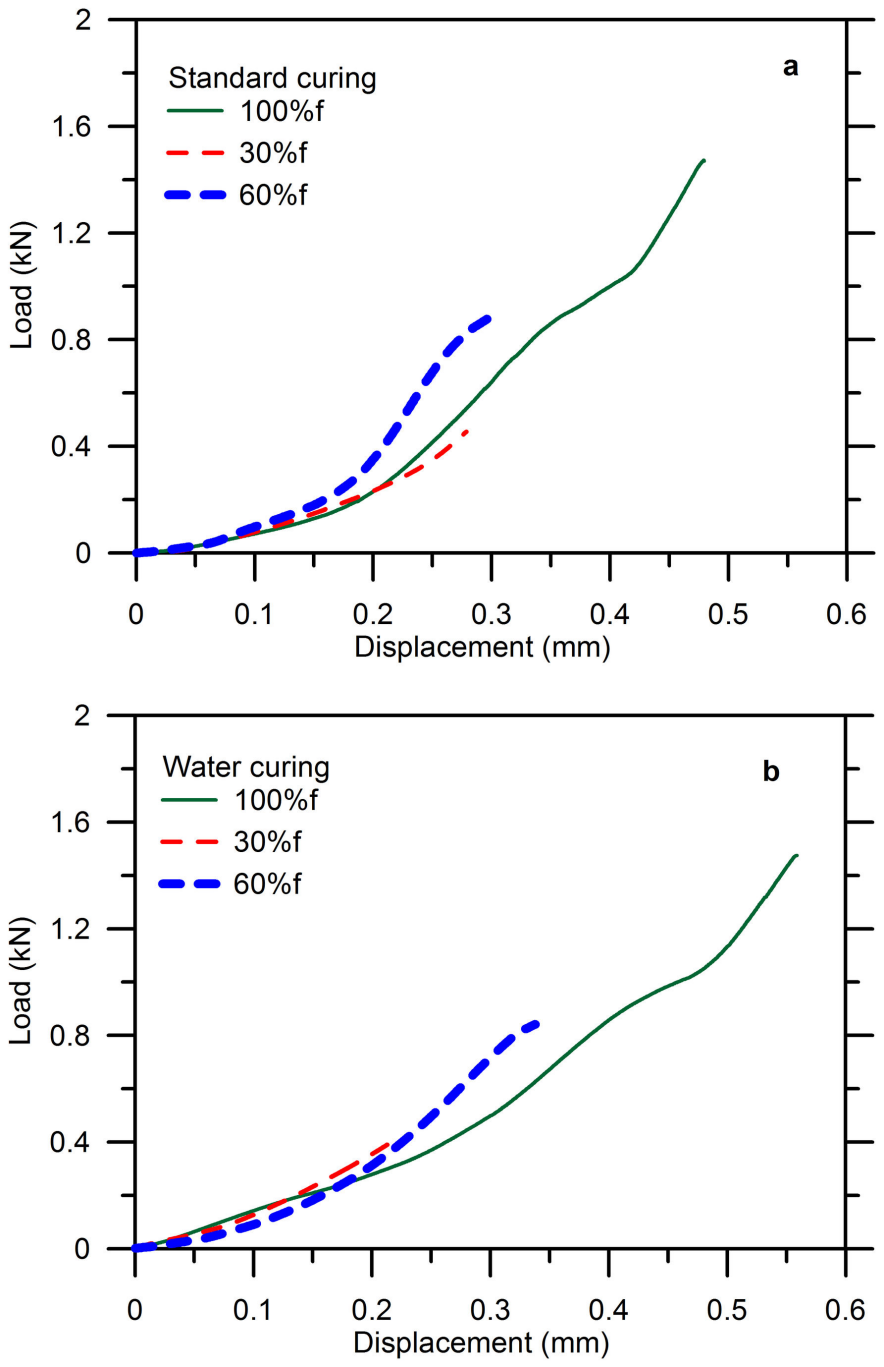
Load-displacement curves for blank cement paste during pre-damage: (a) standard curing (b) water curing.

Figure 5 shows the load-displacement curve of the specimens without microcapsule when the cracks were fabricated by various levels of loads as 30%, 60% and 100% of the maximum resistance, aged in air (a) and water (b) respectively. The reason that these three levels were chosen can be illustrated by that the failure of cementitious material is a process of initiation of microcracks, stable growth and propagation until coalescence of cracks to fracture the material due to its complex microstructure, which normally corresponds to 30%, 50%~60% and 75%~100% of its strength respectively, according to a quasi-linear constitutive relationship of stress-strain for concrete in compression [54]. While for uniaxial tension, the stress-strain curve keeps straight for a longer time until 60% of the ultimate stress, because the tensile stress is not beneficial to render cracks than that of compression [54]. Unlike concrete, cement paste is more elastic and the stress-strain curve maintains linear related until 80~90% of its resistance. But the geometry and amount of cracks are assumed to be quite different for the three levels of pre-damage. 

Normal cementitious materials are generally assumed to act as linear elasticity within 30% of its strength considering cracking is minimal in this stage, after which the linear proportion between load and deformation may appear to diverge [54]. This agreed well with the results presented in Figure 5, where the curves were basically coincident for all the loads. . Moreover, the load-displacement curve began to deviate from a straight line with the increasing load. The specimens were pre-damaged with 60%*f* during which cracking and plastic flow potentially occurred and contributed to a nonlinear and inelastic mechanical behavior of the cementitious composites [54]. The deviation between the curves was due to the fact that only one curve for each type of condition is presented and so is the following.

It is noteworthy that the defected specimens by 100%*f* were weakened due to both cracking and crushing while the pre-damaged specimens by a lower load were mainly weakened by cracking. Thus, the methods of repair of defected specimens and pre-damaged specimens should be different, depending on the type and size of damaged area [55]. 100%*f* has lead to damage localization and crush of the specimen when it was flexural loaded and thus no events of healing or repair by the epoxy were supposed to take place following. While in compression, the test piece was considered to have failed when the internal cracking has reached such an advanced state that the specimen was unable to carry a higher load even though no signs of external rupture were visible [54]. Therefore, healing or repair was still considered to potentially take place in these specimens, but need other methods more, such as fully wrap, spiral strap, or district strap with some flexible sheets to strengthen the defected structure [55]. 

**Figure 6:**
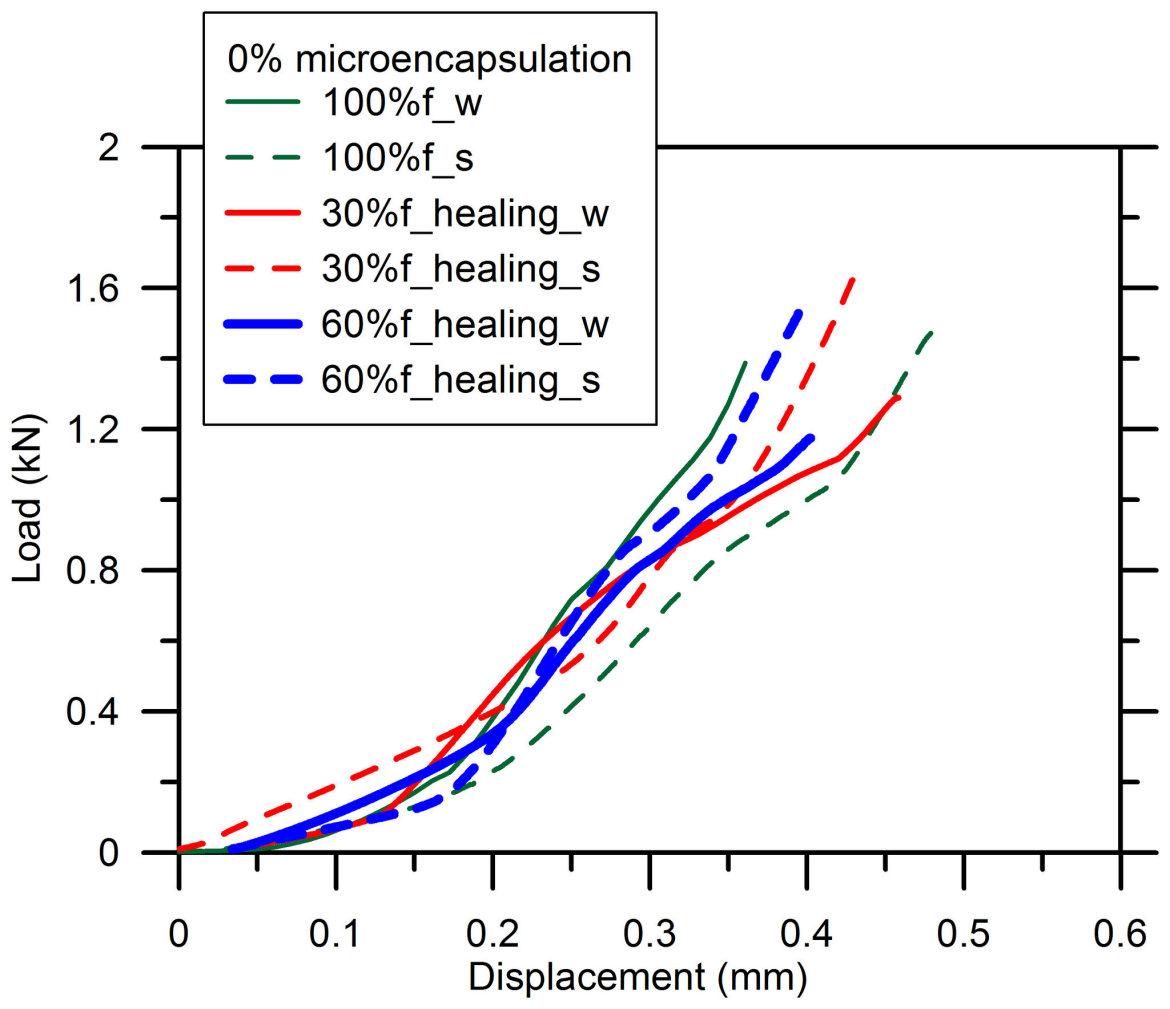
Reloading-displacement curves for blank cement paste after healing.

Figure 6 shows the results for the specimens without microcapsule which were firstly loaded by 30%*f*, 60%*f* and 100%*f* to trigger cracks and followed by 1d of aging for cracks healing, both in water and air conditions, except the failed one at 100%*f* as aforementioned. Generally, the curves for the specimens before and after healing were very close to each other, irrespective it was air or water stored. Even the specimens pre-damaged by a higher load did not show that much difference on load-displacement curve for both conditions. Since no microcapsules were incorporated, no healing efficiency was supposed to be seen. Though it has been reported that a further hydration of unhydrated cementitious components is a potential healing mechanism when the new exposed surfaces of those particles by cracking is accessible to water, it is a minor possibility in such an old cement paste without any supplementary additives [11,56]. Moreover, the healing conditions seemed to not show a noticeable influence on the mechanical performance of the specimens except the fractured which exhibited a smaller deformation at the same load applied when the specimen was water-stored. This can be attributed to a more complete hydration of cement which accelerated the growth of strength when water was sufficiently available. 

**Figure 7:**
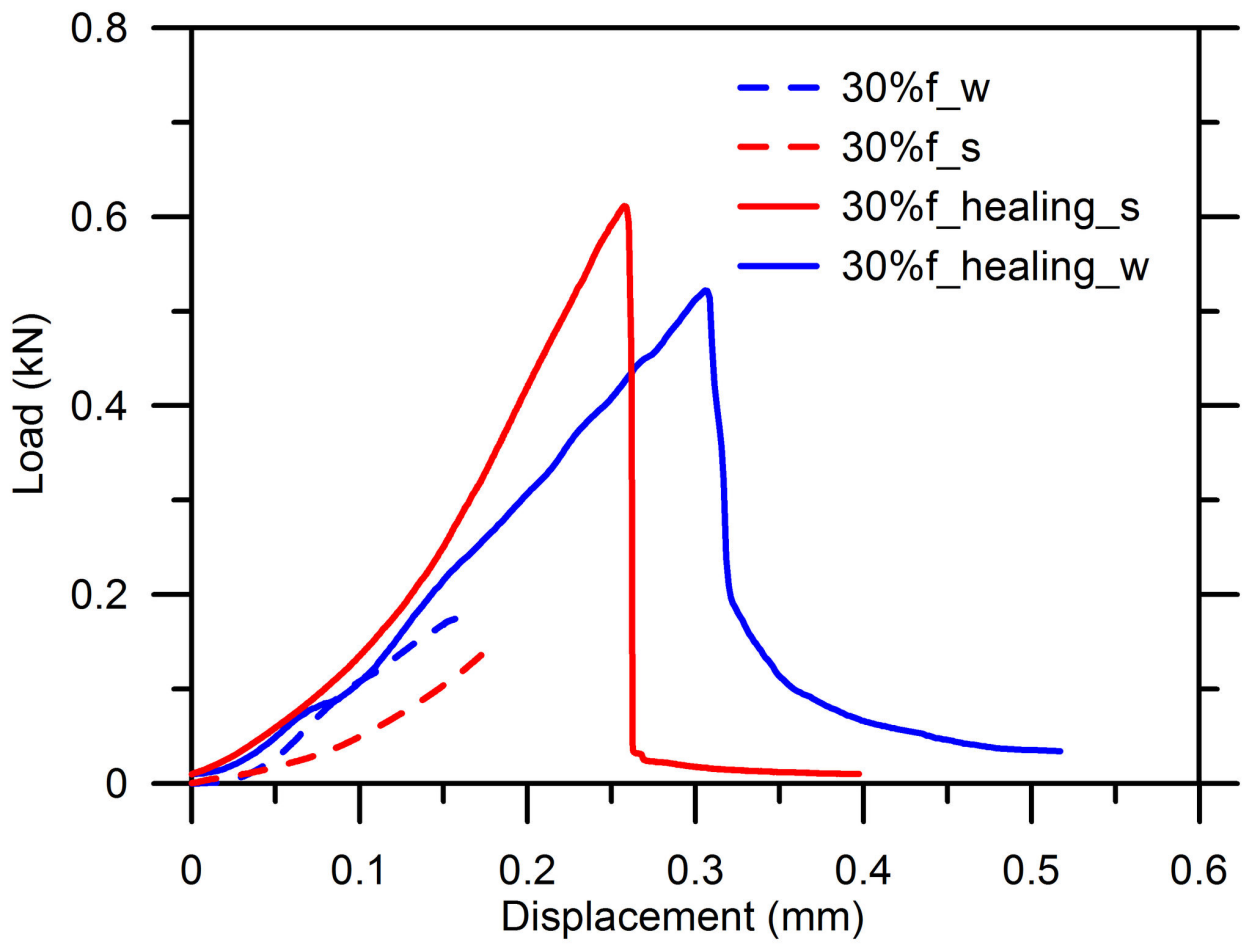
Reloading-displacement curves for cement paste with 1% of microcapsules after healing.

Figure 7 shows the result for the specimens with 1% of microcapsules before and after healing when the specimens were stressed until 30%*f*. The water-cured one exhibited a higher stiffness than that aged in air due to a more sufficient hydration of cement, which is consistent with the results when no microcapsules were added, as shown in Figure 6 (see 100%*f*). 

A higher stiffness was obtained from the reloading curve (slope of the red solid line) compared to the pre-damage profiles (slope of the red dash line) when the specimens were standard cured. But this cannot be concluded as an improvement of the stiffness due to the healing effect of the adhesive because cracks have been validated to be stable and minimal within the elastic range [54]. Thus, a little amount of the epoxy was released upon breakage of the microcapsules to fill the gaps; besides, the sparse healing cracks did not affect the stiffness very much, which is not sensitive enough to the few local cracks but more dependent on the bulk properties of material [57,58]. Additionally, the water-cured specimens did not act like this as the reloading curve was very coincident with the first loading curve as highlighted by the blue lines. A further study is needed to find the reason for the improved stiffness of the specimen aged in air.

However, the load-displacement behavior after healing became very close to each other for the specimens until 0.2kN, after which the specimens aged in air reached to about 0.6kN in the maximum resistance with respect to 0.25mm in displacement, while the specimens stored in water displayed a shift to a lower strength but a greater displacement shown in Figure 7. In addition, a more closed investigation on the improvement of strength following validated that this can be partially attributed to the refined cracks by polymerization of the epoxy. Furthermore, the flexural strength is quite sensitive to the microcracks that lead to stress-intensive locally. Stress can still transfer when the gap was filled by an adhesive that is strong enough to bridge the two apart faces. Furthermore, the sharp tip of the cracks might alter after healing so that the stress field in the vicinity of the original cracks redistributed as well [59]. Because all of these, an improved fracture behavior can be expected for the healing specimens.

**Figure 8:**
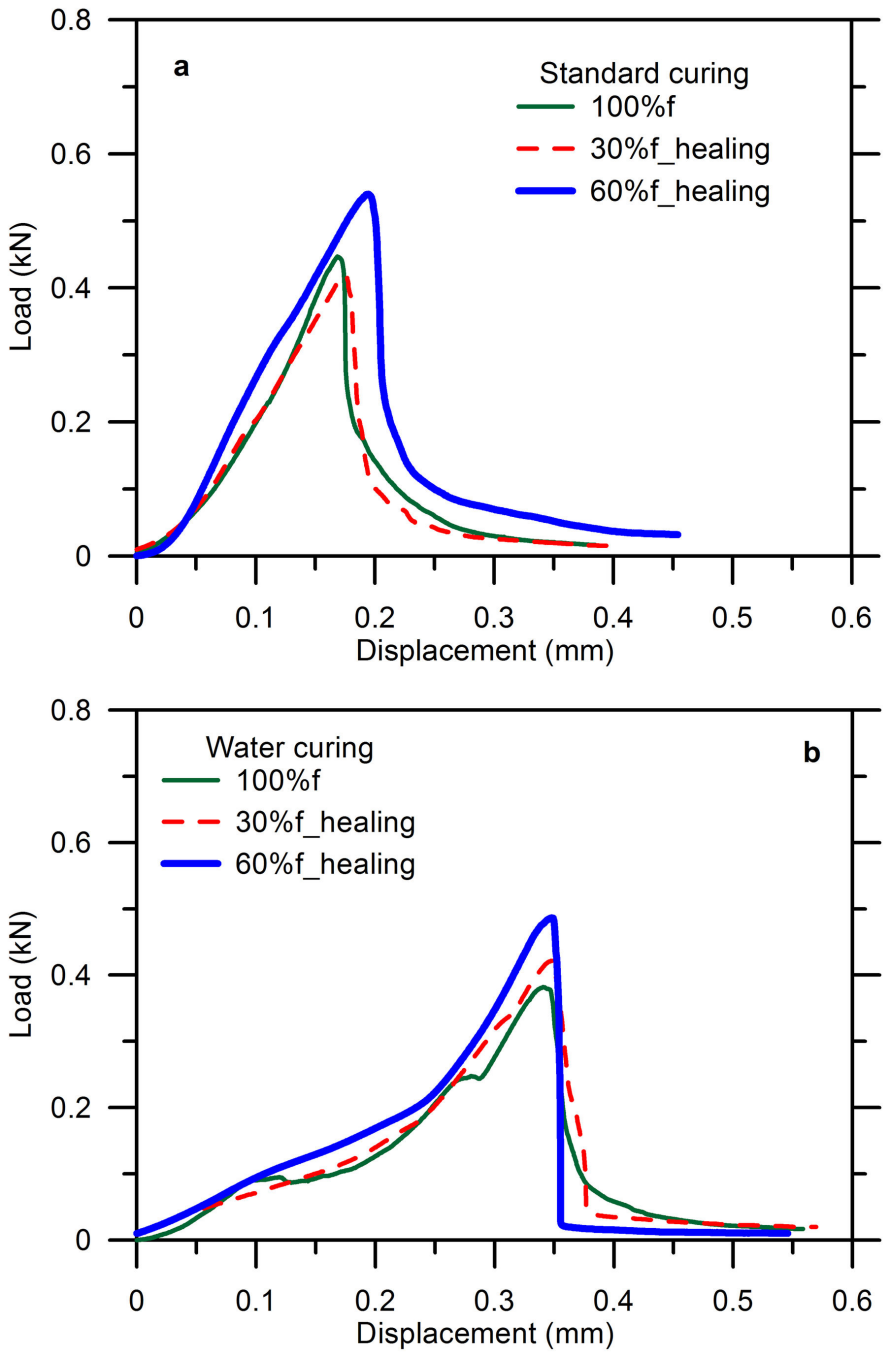
Reloading-displacement curves for cement paste with 2% of microcapsules after healing: (a) standard curing (b) water curing.

Figure 8a shows the results for the specimens cured in air with 2% of microcapsules. The reloading curve of the specimens pre-damaged by 30%*f* was very close to the intact one without allowed healing until failure (labeled as 100%*f*). When the specimens were prior damaged by 60%*f*, followed by 1d of aging, the healing efficiency was noticeable as seen from a higher strength and a higher fracture energy that is the area covered by the reloading-displacement profile as highlighted by the blue line. It has been known that the area under the constitutive curve represents the total fracture energy that is required to break the specimen [60]. Thus, the improved curve after healing demonstrated that more energy was needed to rupture the healing specimen because of the epoxy that provided a sufficiently strong bond between the exposed cracks and thus forced other new cracks to open, in order to prevent the reopening of the reattached cracks [18]. In comparison with autogenic healing of cementitious composites by crystallization of mineral additives, CaCO_3_ in most cases, no gain in mechanical performance has been obtained [22,25]. 

Figure 8b shows the similar results for the specimens aged in water. A recovery of strength has been seen for all the specimens with the various levels of damage. The specimen pre-damaged by 60%*f* had the highest strength restoration of 0.5kN compared to 0.4kN for the reference that was loaded up to failure directly without healing following. 

**Figure 9:**
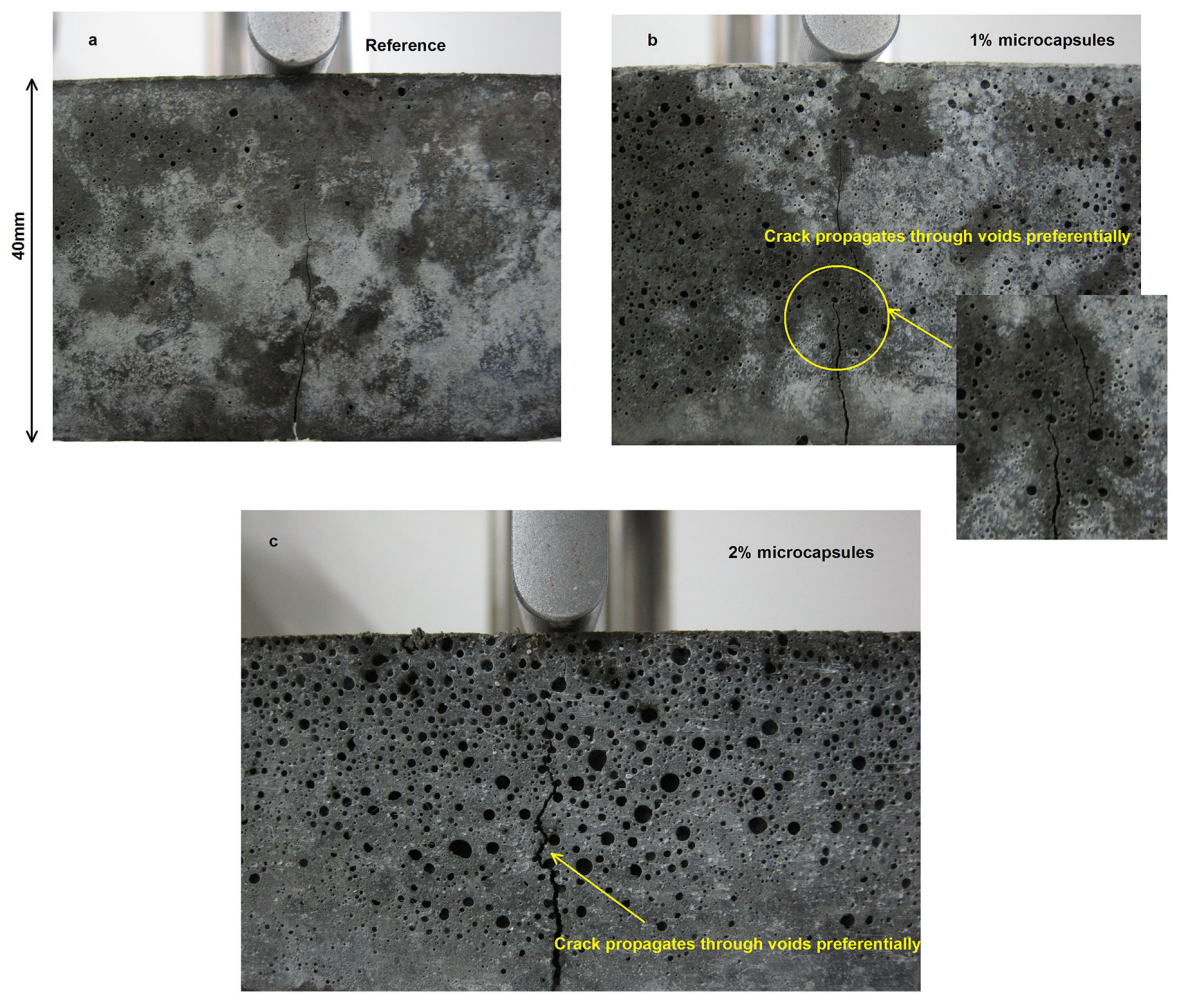
Crack penetration of specimens before failure: (a) reference (b) 1% of microcapsules (c) 2% of microcapsules.

It should be noted that the post-peak regime can be detected once the microcapsules were added whereas the blank specimens always showed such an abrupt brittle failure that the post-peak regime was unable to be captured. This can be explained by that the more voids, which were produced by the residual surfactants in the emulsion of microcapsule, contributed to a more energy dissipation for arresting and shifting of the cracks that propagated towards the voids preferentially as shown in Figure 9 [61]. Besides, it is possible that the crack could be blunt by the voids on the path of propagation to reduce the stress concentration around [18], provided the energy released by the formation of new surfaces is not strong enough to overcome that dissipated or relaxed energy by the voids. Moreover, the much higher tensile resistance of epoxy than that of cementitious composites also strengthened the flexural performance of the specimen. 

**Figure 10:**
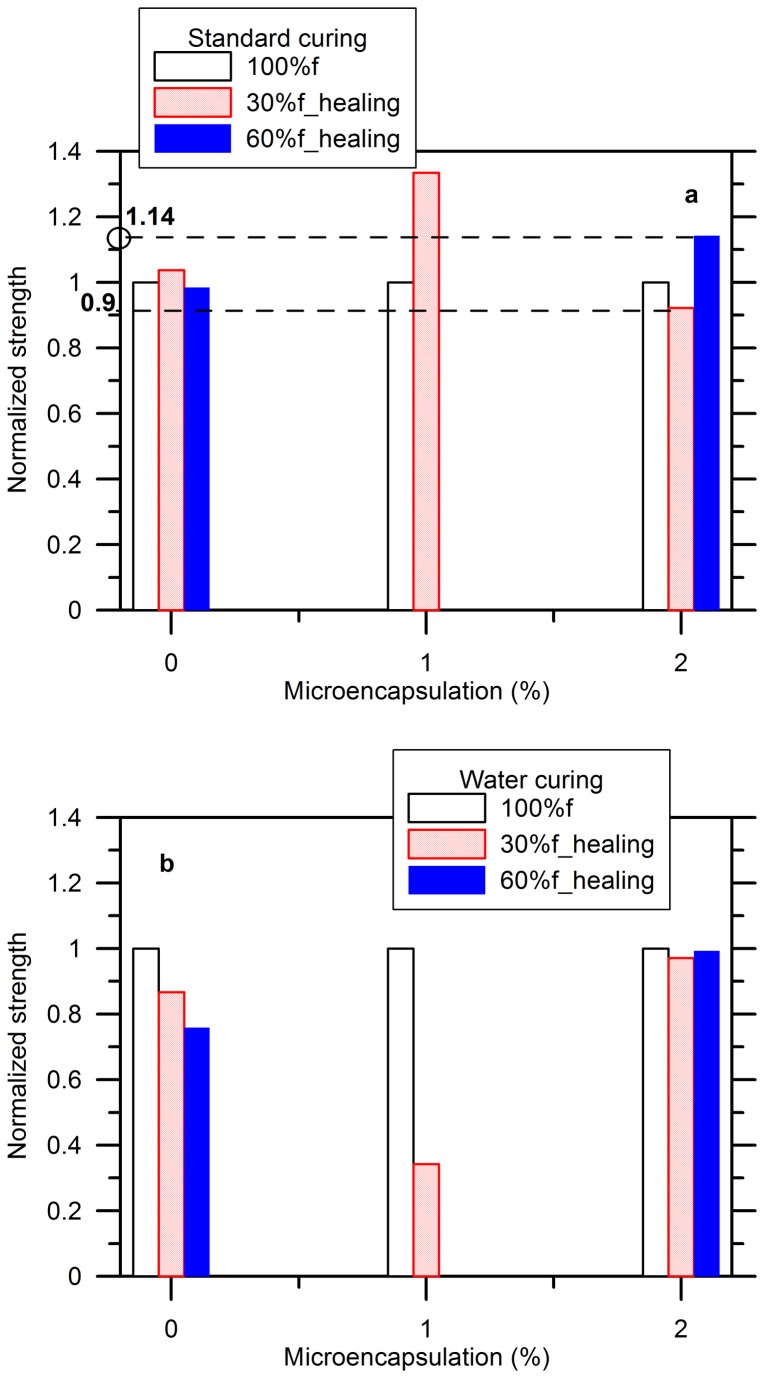
Influence of content of microcapsules on flexural strength restoration of cement paste: (a) standard curing (b) water curing.

Figure 10a shows the flexural strength for the damaging specimens with their respective recovered strength. The normalized strength recovery is reported by the ratio of the maximum strength reached after cracks healing to the maximum strength of the reference in the initial state to remove the influences of the inherent flaws, defects, and size of the specimens. The data is missing for the specimens with 1% of microcapsules and 60% pre-damage due to being incidentally broken. The strengths of the blank specimen before and after healing were very close to each other. When 1% of microcapsules were added, the healing strength reached to 1.3-1.4. The specimens with 2% of microcapsules gained 1.14 times of strength restoration after 60%*f* was applied, but this value was 0.9 with respect to 30%*f*. This can be explained by the fact that a higher stress can lead to a more deteriorated specimen as characterized by the bigger cracks in geometry, which are easier to break the shell of a capsule when propagation to release the epoxy occurred and followed by it flowing into the cracks due to a capillary effect. It should be noted that the emulsion weakened the strength of the specimens because of the voids generated by the surfactants shown in Figure 9, where the specimen with a higher content of microcapsules exhibited more and bigger voids on the out surfaces. Therefore, the healing strength depends on the dual effect of the emulsion with capsules: compromising the strength and the healing capability.

**Figure 11:**
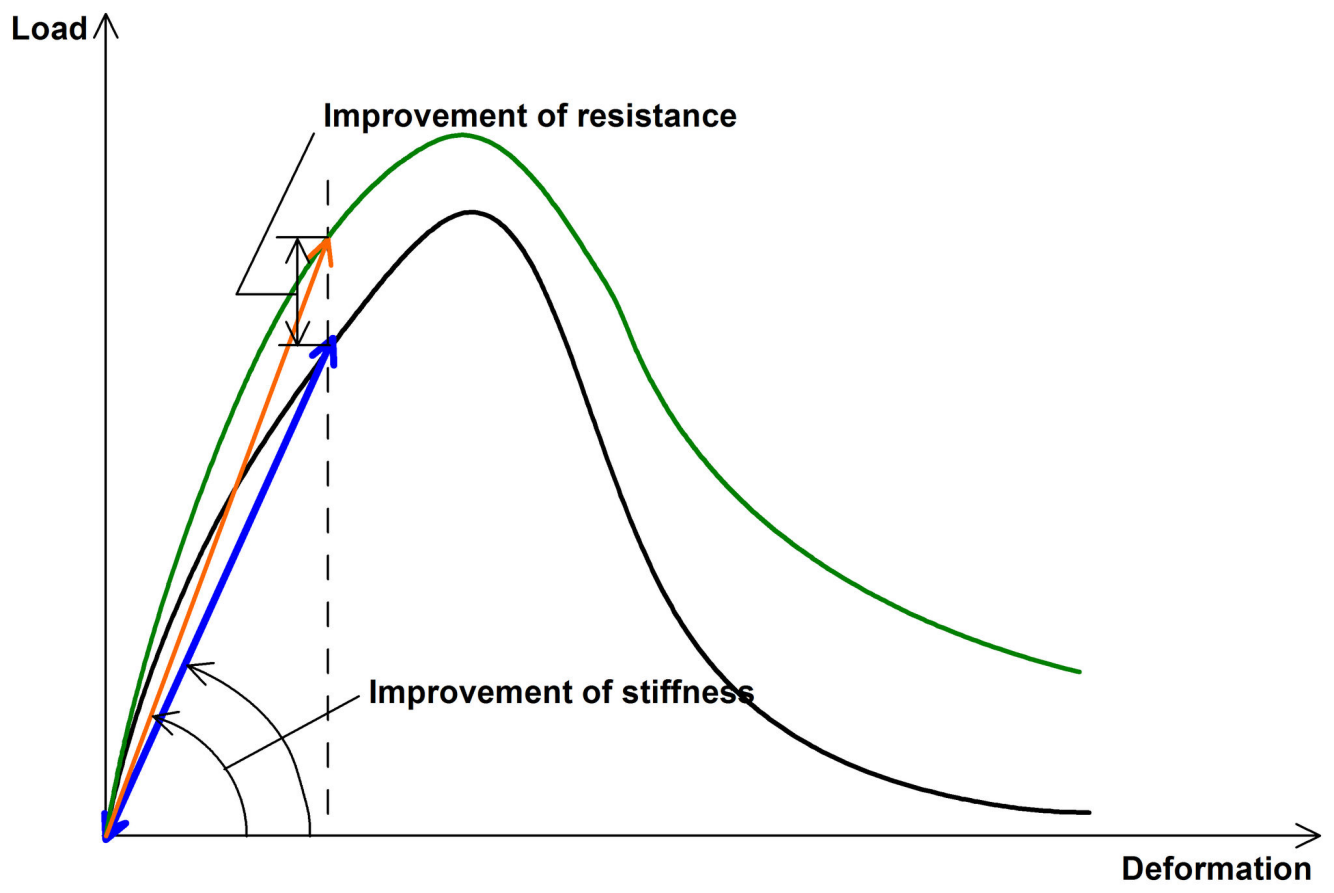
Schematic load-deformation relationship of cementitious material.

Figure 10b shows the same results for the specimens stored in water. The normalized strength decreased with the increasing degree of damage for the blank specimen whereas this value kept about 1 for all the damages when air-cured. This is possibly due to the sensitivity of the flexural strength to the intensive stress induced by defects, flaws, etc. A typical constitutive relationship of cementitious material is schematically shown in Figure 11, where the quasi-linear proportion between load and deformation makes the reloading path coincident with the unloading path at the pre-peak stage as highlighted by a blue solid line. When the cracks are bridged by a healing adhesive like epoxy, which is normally with a higher strength than that of cementitious substrate, the stiffness can improve as demonstrated by that the reloading path will follow the green line. The load resistance will increase accordingly with respect to the same deformation. Any intervention in cracks can lead to a big deviation of the flexural behavior since its failure is tension-dominated, which is highly sensitive to stress concentration. Apparently, the strength recovery was low for the case of 1% of microcapsules and 30% pre-damage. More study is ongoing to find the reason. Moreover, the normalized strength kept at 1 when 2% of microcapsules were added due to more cracks healing. Thus, it seemed that the specimen with 2% of microcapsules and 60% pre-damage exhibited the best healing effect in terms of flexural resistance according to the combined results of the two conditions.

**Figure 12:**
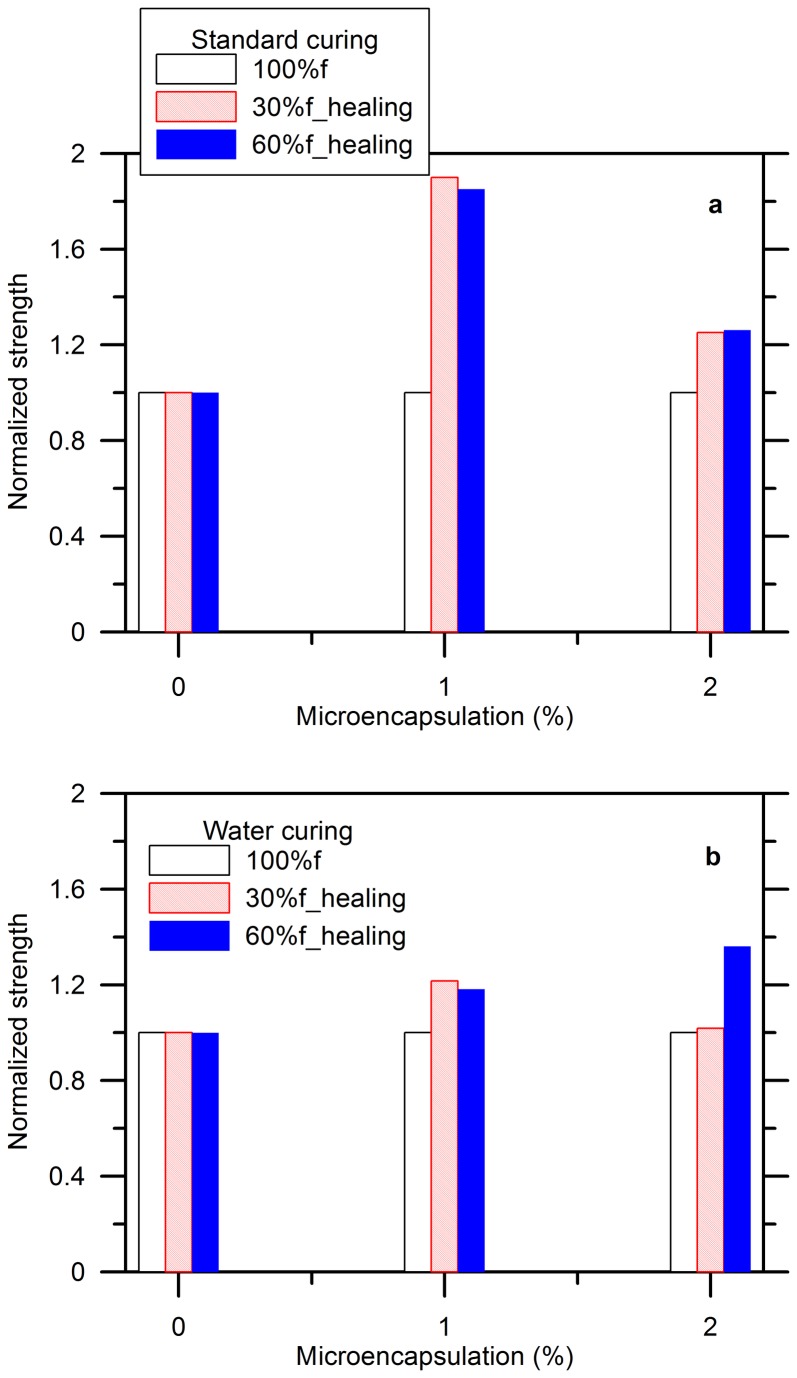
Influence of content of microcapsules on compressive strength restoration of cement paste: (a) standard curing (b) water curing.

Figure 12 shows the compressive strength of the flexural fractured specimens. Compared to the fluctuant flexural strength shown in Figure 10, the normalized strength remained at 1 for the blank specimens, irrespective of the curing conditions. This was caused by that compression, similar to stiffness, being more dependent on the bulk properties of materials as it is an operation to render cracks. In Figure 12a, a good strength restoration was seen for the specimens stored in air with 1% of microcapsules. The healing compressive strength reached to 1.8 for both 30% and 60% levels of damage. It did not show that much strength restoration when the capsules increased to 2%. A best healing efficiency can only be expected when the amount and dimension of cracks fit the released healing agent upon breakage to fill in those cracks perfectly and completely. Either a too deteriorated cement paste, which left a lot of unhealed cracks inside, or too many microcapsules, which introduced an amount of voids produced by the surfactants and/or left by the exhausted capsules resulted in a poor healing efficiency. Moreover, the level of damage did not show a remarkable influence on the compressive strength recovery in the present study.

The specimens kept in water also showed the strength recovery to a certain extent when the capsules were added (Figure 12b). The normalized strength reached to 1.2 for the specimens with 1% of capsules. The strength did not improve when the capsules increased to 2% and the specimen was pre-cracked by 30%*f* ; however, it gained to 1.4 times for the reference in strength when the load was raised to 60%*f* compared to 1.2 due to standard curing, as shown in Figure 12a. This might be related to the hardening of epoxy, which can be affected by the presence of water or the humidity around. A further investigation is in process to make a better understanding about it.

Generally speaking, the specimens aged in air showed a better strength restoration when 1% of capsules were added whereas the water-cured specimens with 2% of capsules showed a better strength recovery after 60% pre-damage was induced..

**Figure 13:**
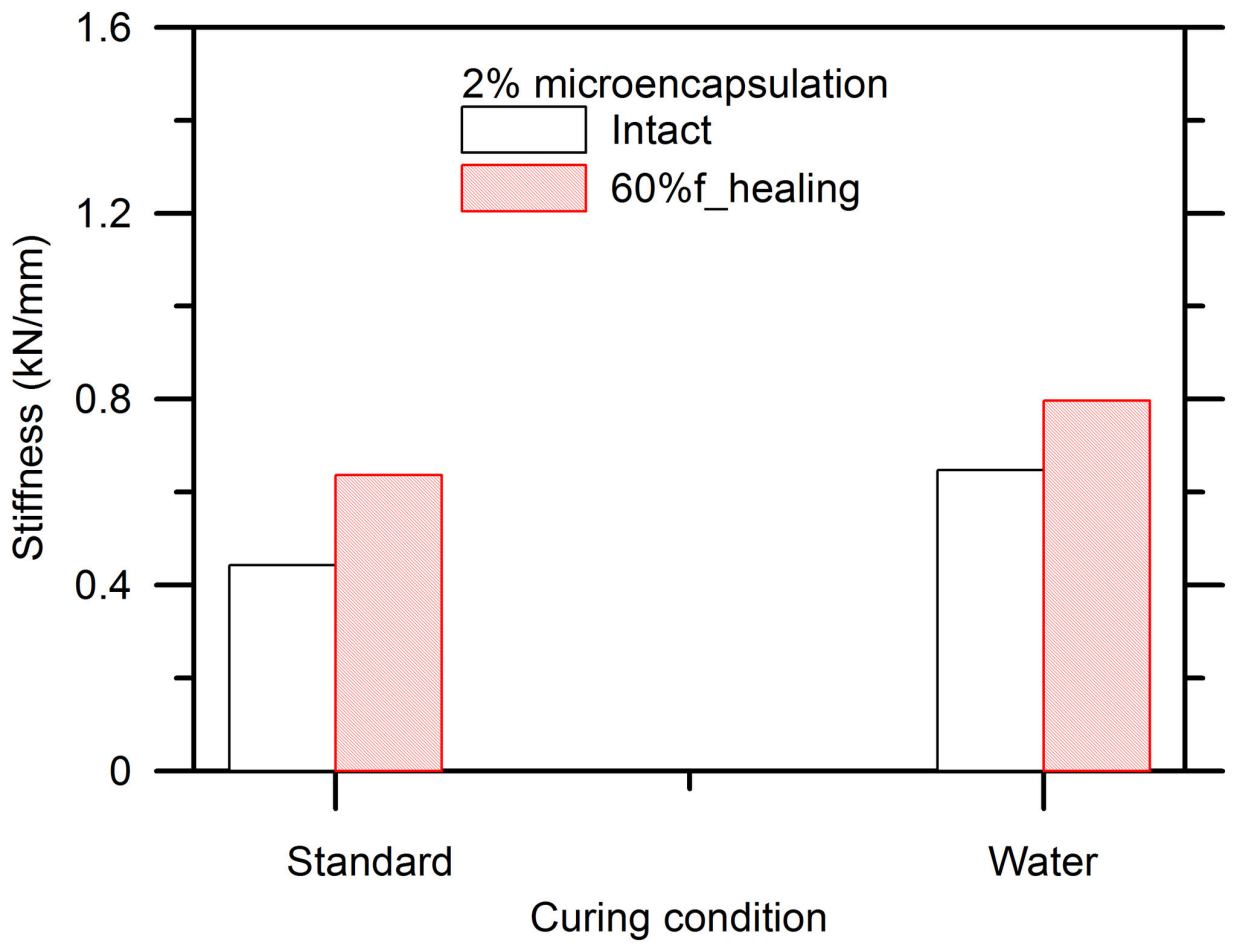
Effect of curing conditions on stiffness recovery of cement paste with 2% microcapsules and 60% pre-damage.

Figure 13 compares the influence of curing condition on the stiffness, which is defined as the slope of the load-displacement curve at 30%*f*, of the specimens containing 2% of capsules and being pre-cracked by 60%*f*. The specimens exhibited a higher stiffness when water-stored as a result of more complete hydration of cement. A slight recovery of stiffness was seen in both conditions.

It is noteworthy that using force operated on the specimen to characterize the degree of damage is controversial and the same ratio of load does not produce the same characteristics of microcracks like size, geometry and amount [62]. Additionally, the specimens cured in water were supposed to be with fewer cracks to be filled due to a more compact microstructure than that aged in air.

**Figure 14:**
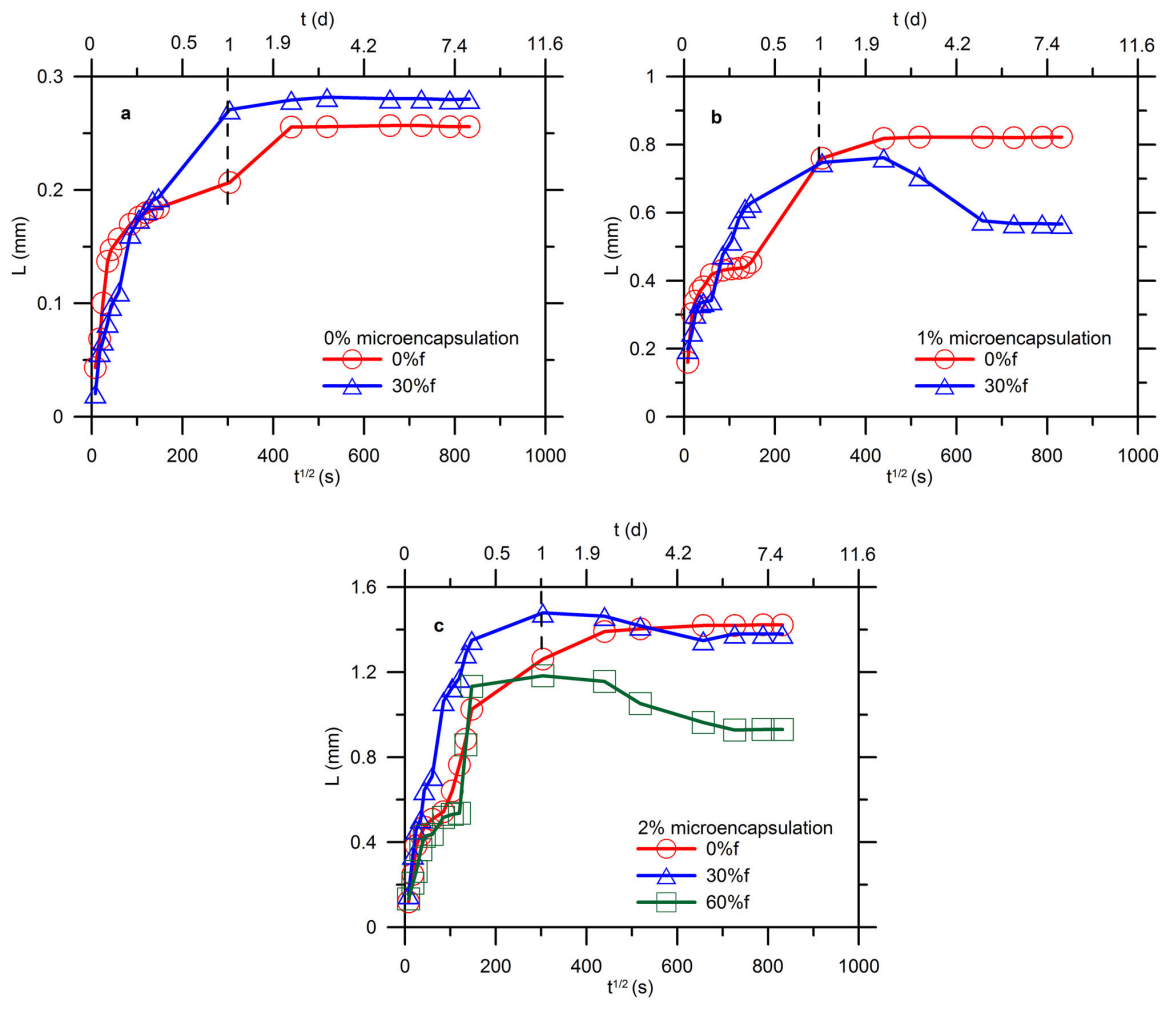
Water sorption of cement paste during healing: (a) reference (b) 1% of microcapsules (c) 2% of microcapsules.

Figure 14a illustrates the water absorption results for the blank specimens acquiring 0% and 30% pre-damage. The water absorbed is plotted as a function of square root of time on the lower x-axis whereas actual time is shown on the upper x-axis. The specimens showed a similar sorptivity (slope of the curves) and amount of absorbed water initially; however, over time the damaging specimens absorbed more water. This occurred because the smaller capillary pores created a higher suction force as outlined by Kelvin-Laplace equation (Eq. 1). The microcracks induced by load are normally bigger than the existing capillary pores in size but the tortuous path of the cracks can still create a capillary effect for water suction. Therefore, the water absorption initiated capillary pores within the first 6h and followed by the microcracks due to stress. The overall effect was a higher rate of water absorption and a high volume of absorbed water for the damaging specimens since 1d immersion in water. The water sorption was progressively to saturate the specimen with elapsed time as the sorption curve became leveled off after 2d. 

$$\ln\left(RH\right)=\frac{2\sigma V_{m}}{r_{m}RT}$$ Eq.1

where: *RH* is the relative humidity, σ is the surface tension of water or pore solution, *V*_*m*_ is the molar volume of water, *r*_*m*_ is the average radius of curvature, *R* is the universal gas constant, and *T* is the absolute temperature.

Figure 14b shows the results for the specimens containing 1% of capsules. The intact specimens exhibited the similar sorption and the rate decreased dramatically after 1d. For the specimens acquiring 30% pre-damage, the water sorption became quite different from that of the reference. The sorptivity and amount of water accumulated displayed the similar increase within the first 6h after which the damaged specimens absorbed water much faster until 1d than that of the intact specimens. More interestingly and importantly, the sorptivity of the damaging specimens started to decrease gradually and then a sharp drop was seen at 4d to a stable value. This can be attributed to that the microcracks induced by stress created a capillary force to accelerate the suction of water following water sorption of the capillary pores within the first 6h. However, the hardening of epoxy released by the capsules took place since it met the hardening agent which was prior added in the mixture when casting. It took about less than 1d to finish polymerization to gain its sufficient strength, which agreed well with the moment of the sorptivity turning to a decrease. Water sucked in can be squeezed out when the adhesive filling the cracks and voids. Thus, the measurement of water sorption validated the self-healing process of the damaging specimens, during which cracks healing contributed to cut off and block the ingress of water in the host matrix. It is noteworthy that using water sorption to characterize the network of microcracks and voids only works on small scales because the capillary force is necessary all the time as a driving force for the suction of water [57,58]. When the cracks are so large that the capillary force is minor, the other external intervention is required instead, such as a pressure of fluid or air to push water through the cracks. 

Figure 14c shows the results for the specimens with 2% of microcapsules. Similarly, the amount of absorbed water and sorptivity of the damaging specimens started to decrease after 1d of aging; however, the accumulated water was more than that containing 1% of capsules, as shown in Figure 14b. This was caused by the more voids that produced by the surfactants as aforementioned. Moreover, 60% of fabricated damage resulted in less absorbed water due to more cracks healing by the epoxy. The water sorption results agreed well with the strength restoration.

**Figure 15:**
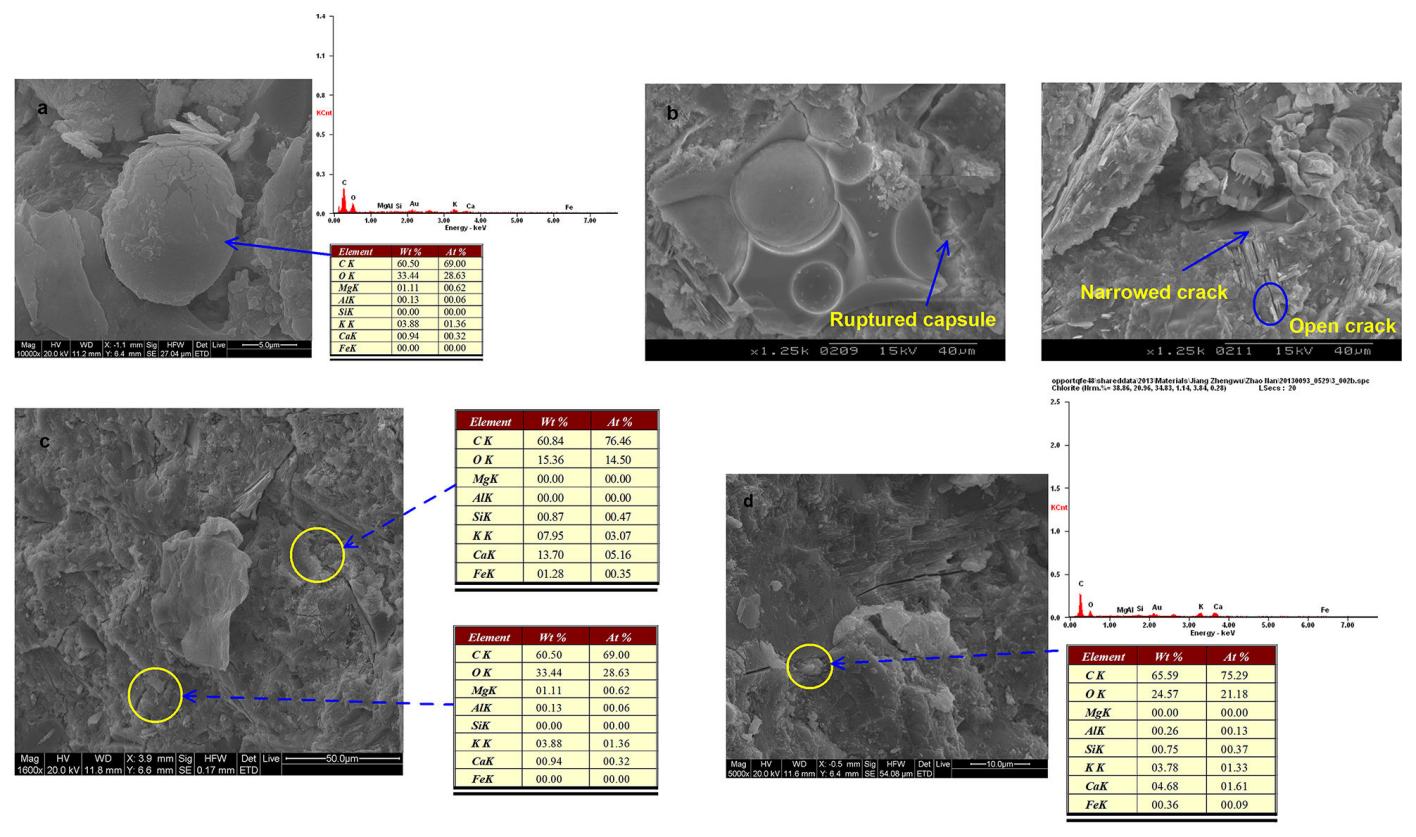
SEM-EDS micrographs of damaging specimens at different stages: (a) 30%*f* (b) 60%*f* (c) capsules layout at 100%*f* (d) healing cracks at 100%*f*.

Figure 15 shows the morphological appearance of microcapsules in the different stages of loading using SEM-EDS. In Figure 15a, a microcapsule with a smooth membrane shell was observed in the specimen loaded by 30%*f*. A network of microcracks was clearly about to spread on the upper part of the capsules. The elements of C and O accounted for 94% by mass according to EDS. When the load increased to 60%*f*, the capsules were open much more, as shown in Figure 15b, where a crack propagated through a capsule completely. By a closer observation, the crack in the vicinity of the capsules was narrower than the other length of the crack. EDS results showed that C and O were still the dominated elements in those narrower locations but not found elsewhere. This implied that the healing agent, epoxy in the current study, was released from the capsules when its shell wall was ruptured by propagating cracks, and filled in the gaps driven by a capillary effect and gravity [17,53]. The nearby cracks were gradually closed with the hardening of the polymers. Therefore, stress can still transfer between the bridged crack surfaces as characterized by the mechanical recovery as described previously. This healing process has also been validated by the measurement of water absorption. When the specimens were failed due to 100%*f*, the capsules layout was detected shown in Figure 15c. The healing cracks were found in the regions close to the capsules as highlighted by the circles in Figure 15d, where the combined proportion of C and O accounted for 90% in total.

## Conclusions

The present work experimentally assessed the healing efficiency of cementitious composite by St-DVB microcapsules enclosing epoxy resin as the healing agent.. The mechanical performance was studied using cement paste specimens that were pre-damaged by flexural load and compressive load following to stimulate the localized and distributed cracks respectively. The effects were considered from varying the content of microcapsules, the curing condition and the level of pre-damage. A test of water absorption was successfully proposed to monitor and characterize the evolution of crack networks during the healing process. The main conclusions can be drawn as follow:

1. The mechanical restoration of cement paste was achieved after cracks healing in both standard and water curing conditions. The specimens aged in air showed the best strength recovery for the case of 1% of microcapsules and 30% degree of pre-damage. While the specimens aged in water showed the best mechanical recovery when 2% of microcapsules were added and 60%*f* was applied. The effect of curing conditions can be attributed to a more complete hydration of cement and compact microstructure accordingly by water curing, which increased the stress required to trigger cracks to break the capsules.
2. No noticeable improved stiffness was seen due to that stiffness is not sensitive enough to the local minor cracks but more dependent on the bulk properties of material as contrary to the flexural strength that can be greatly affected by the local cracks. Whereas the total fracture energy to rupture the specimens increased due to a sufficiently strong bond between the exposed surfaces by the epoxy; besides, the voids produced by the residual surfactants contributed to not only arresting and shifting of the cracks but also reducing the stress concentrations around.
3. The test of water sorption was validated to be an effective method to monitor and characterize the healing process, involving releasing, gradual filling, and hardening of the epoxy, which contributed to cut off and block the ingress of water in cement paste.

It should be noted that the voids, either left by the exhausted capsules or generated by the surfactants, can decrease the strength of the specimens. Therefore, the resulted strength depends on the dual effect of capsules: compromising the strength and the healing capability. 

Moreover, using water absorption to characterize the network of microcracks and voids only works on so small scales that water is mainly driven by capillary force to be absorbed. When the cracks are so large that the capillary force is negligible, the other deliberate external intervention is required, such as an applied pressure of fluid or air to push water penetrating through the cracks. In these cases, the single measurement of water absorption is not effective enough to characterize the crack system anymore.
